# Phenology overshadows seed treatment and cultivar effects on fall armyworm gut microbiome following short-term feeding on rice

**DOI:** 10.7717/peerj.20458

**Published:** 2026-01-20

**Authors:** Devi Balakrishnan, Stephanie Cromwell, Paul A. Ayayee, Nick Bateman, Rupesh Kariyat

**Affiliations:** 1 Department of Entomology and Plant Pathology, University of Arkansas, Fayetteville, AR, United States of America; 2 Department of Biology, University of Nebraska—Omaha, Omaha, NE, United States of America

**Keywords:** Herbivory, Defense, Phenology, Illumina, Seed treatment

## Abstract

Plants mediate host susceptibility or resistance to infesting insects through various documented ways, and possibly via disrupting insect gut microbiota, an area that is underexplored in rice. The fall armyworm (FAW) (*Spodoptera frugiperda* (J.E. Smith) is a highly invasive herbivore that infests ∼350 host plant species, including rice (*Oryza sativa* L.). Exploring the impacts of chemical seed treatments on rice against FAW on the composition and attendant detoxification functionalities of the gut microbiota, as well as the subsequent effect on larval development, could inform the development of more effective management strategies. To test this, we characterized the gut microbiota of FAW 4 th instar larvae exposed to rice cultivars with and without chemical seed treatment (CruiserMaxx plus Vibrance package) at vegetative and reproductive stages for 96-hours. Results did not determine any statistically significant effects of chemical treatments and rice cultivars on larval microbiota composition. However, crop phenology (vegetative and reproductive stages) significantly impacted larval microbiota. Bacterial taxa previously implicated in FAW larval detoxification of plant secondary compounds, as well as FAW growth, and development, were uncovered across larvae. Hence, our findings highlight the importance of considering rice phenology and associated traits in developing management strategies against this highly polyphagous pest.

## Introduction

Gut microbiota (protists, fungi, archaea, and bacteria) play vital roles in the growth and development of insects (Engel & Moran, 2013; Siddiqui et al., 2022; Liu et al., 2023). Among these, bacterial communities make up the majority, that drastically vary in total size, composition, and function (Engel & Moran, 2013). These functions include synthesizing essential nutrients to protection from parasites, predators, and pathogens to degradation of toxins and insecticides (Engel & Moran, 2013; Jing, Qi & Wang, 2020; Mondal et al., 2023). The diverse groups of bacteria involved in these processes vary with diet, host, and life stages of organisms (Yun et al., 2014; Luo et al., 2021). Many gut bacteria have been proven to be involved in the survival of insect hosts, helping them to adapt to adverse environmental conditions (Luo et al., 2021). Understanding the changes in the gut microbiota of insects in response to these factors can be used as a potential tool in developing sustainable management practices (Zhang, Zhang & Xu, 2023; Rupawate et al., 2023).

The fall armyworm (FAW), *Spodoptera frugiperda* (J.E. Smith) (Lepidoptera: Noctuidae) is one of the most destructive and a highly polyphagous insect pest, that feeds on more than 350 host species (Montezano et al. 2018; Balakrishnan, Srivastava & Kariyat, 2024). A major concern about FAW is their adaptive potential to damage diverse host groups through host switching (Fatoretto et al., 2017; Wang et al., 2023), mediated by the ability to detoxify plant toxins and insecticides (Qi et al., 2024; Zhang et al. 2024a; Zhang et al. 2024b; Mahalle et al., 2024). One of the important hosts of FAW is rice (*Oryza sativa* L.) which is the staple food crop that 50% of the global population depends on (Liu et al., 2023; Engel & Moran, 2013; Balakrishnan, Srivastava & Kariyat, 2024). Despite not being considered as a major pest of rice (Xu et al., 2025), the potential of FAW being a global pest of rice is not that far due to changing climatic conditions and rising global temperatures (Yan et al., 2022; Zanzana et al., 2024). Drastic changes in the climatic conditions, especially temperature fluctuations, contribute to shortening the generation time inducing them to be multivoltine (Ramirez-Cabral, Kumar & Shabani, 2017). Moreover, their adaptability and survival traits enable them to withstand most of the current management practices (Chen et al., 2023; Samanta et al., 2023), thereby posing a serious threat to global food security.

Host plant switching by FAW has been reported to be underscored by changes in gut microbial composition. Ma et al. (2024) also reported differences in gut bacterial composition and FAW larval fitness across various plant hosts, including maize, rice and wheat. As the host plants vary in the level of nutrients and toxins, gut microbiota shift and modify their composition to ensure normal growth and development in insects. Hence, diet composition also plays a significant role in fine-tuning the community structure of larval gut microbiota. Furthermore, the quantity and quality of nutrients, metabolites, and phytotoxins vary with plant phenology (Alipour & Saharkhiz, 2016; Gontar et al., 2024), underscoring the importance of incorporating host plant phenological differences into microbiome studies. Overall, intraspecific variation through cultivars and varieties can affect phenology, and primary and secondary metabolites among host plant cultivars, and chemical treatments of rice seeds may be key determinants of FAW larval gut microbiomes and survival. However, very little is known about these aspects of rice-FAW interactions.

A common and effective method for managing FAW is seed treatment with chemicals (Villegas, Wilson & Stout, 2019; Li et al., 2022), which is a preventive measure to protect early crop growth stages (Sharma et al., 2015; Oliveira et al., 2022). However, the rapid insecticide resistance development in FAW, with resistance reported over 45 active pesticide ingredients (Mota-Sanchez & Wise, 2023)- poses a significant challenge in effective pest management. Previous studies have shown that insecticide resistance in FAW can be primarily attributed to the ability of associated gut bacteria to detoxify, sequester, or expel toxic compounds (Gomes, 2024; Tang et al. 2022). For instance, Qi et al. (2024) demonstrated the ability of gut bacteria in FAW to convert the endophytes in maize into their own probiotics to detoxify the defensive metabolite, benzoxazinoids. Similarly, Gu et al. (2024), pointed out the changes in the composition of bacteria with a hike in the population of *Spingomonas,* a symbiotic bacterium in FAW by directly applying the chemical, chlorantraniliprole as a microdroplet on the larval body. They observed that this bacterial group provides protection from the insecticide, and an increase in the population of the bacterium can be considered as a warning indication for early-stage resistance development.

In the current study, we investigated whether the FAW larval gut bacterial community is affected by the seed treatment (CruiserMaxx plus Vibrance package), phenology (vegetative and reproductive stages), and rice cultivars (RT 7301, Diamond, and Jupiter- the former is a hybrid, and the latter are conventional cultivars commonly grown in Southern United States). We hypothesized that reproductive stage and seed-treated rice cultivars will have a lower gut bacterial diversity and altered composition compared to vegetative and untreated cultivars. Furthermore, we anticipated that the cultivars will significantly influence the bacterial community composition of FAW post feeding.

## Materials & Methods

### Plant materials and insect rearing

Seeds of three rice cultivars: RT 7301, Diamond, and Jupiter grown in the mid-south region of the United States were procured from the University of Arkansas Foundation Seed program. The seeds were treated with CruiserMaxx Rice plus Vibrance package, a combination formulation containing both insecticidal and fungicidal components. The insecticide component is thiamethoxam (that affects the insect’s nervous system by binding to its nicotinic acetylcholine receptors (nAChRs) (Insecticide Resistance Action Committee (IRAC), 2025) and fungicide components are Fludioxonil, Azoxystrobin, Mefenoxam, and Sedaxane (that affects signal transductions, respiration, and nucleic acid metabolisms (Fungicide Resistance Action Committee (FRAC), 2024)) (CruiserMaxx Rice rate: 4.375 g/kg of seed; Vibrance rate: 0.075 g/kg of seed), with insecticide compound: thiamethoxam (142.2 g ai/kg seed), and fungicides compounds: Fludioxonil (1.5 g ai/kg seed), Azoxystrobin (7.17 g ai/kg seed), Mefenoxam (8.83 g ai/kg seed), and Sedaxane (4.07 g ai/kg seed). Both seed-treated and untreated plants were grown in Deepot tree pots (6.9 cm diameter, 20.3 cm deep, Greenhouse Megastore, Danville, Illinois, USA) in LP15 Pro-mix potting soil (Farmers Co-Op, Van Buren, Arkansas, USA) in green house (28 °C–30 °C temperatures and ∼70% humidity). The plants were fertilized with Osmocote Plus (15-9-12) one week after germination and drenched with iron chelate at fortnightly intervals. Extra attention was taken to keep the deepots water saturated over the course of the experiment (Balakrishnan, Srivastava & Kariyat, 2024). We selected two growth stages, vegetative stage (V6: Collar formation on leaf 6 on main stem) and reproductive stage (R3: Panicle exsertion stage where tip of panicle is above collar of flag leaf) for the study.

FAW eggs were purchased from Frontier Agricultural Sciences, Newark, Delaware, USA and allowed to hatch in the laboratory. Neonates were fed with a wheat-based germ diet till fourth instar, (Product Code: F9772; Frontier Agricultural Sciences, Newark, Delaware, USA) made as per our previous works (Balakrishnan, Srivastava & Kariyat, 2024; Balakrishnan et al., 2025; Vasquez et al., 2024; Ayala et al., 2024). Fourth instar larvae were then used for the experiments.

### FAW exposure to rice

The larvae were exposed to three cultivars at two different phenological stages: V6 and R3, for 96 h by enclosing each larva in an organza bag (10.2 cm × 15.2 cm, Volcanic, Amazon, Seattle, Washington, USA). After 96 h, the larvae were collected and preserved at −80 °C separately in two mL Eppendorf tubes for DNA extraction. A total of 20 larvae per cultivar were used for DNA analysis and quantification.

### DNA extraction, sample processing, and Illumina sequencing

Larvae were surface sterilized by immersing in a 1% detergent solution (Micro-90, Capitol Scientific, Inc., Austin, TX, USA) for 1 min, followed by two immersions in deionized water for one minute, respectively, prior to dissection and removal of entire alimentary system. DNA was extracted using the DNeasy Powersoil Pro kit (Qiagen Inc. Germantown, MD, USA). Universal bacterial primers 27F and 1492R were used to validate the microbial fraction of the larval DNA (Frank et al., 2008). High-throughput sequencing was then performed for all samples at the Genomics Core Facility, University of Nebraska Medical Center. Amplicons targeting the V4 (515-F) and V5 (907-R) variable regions (Rintala et al., 2017) were generated. The concentrations of DNA were then measured with the Qubit 3.0 (Qubit™, Thermofisher, Waltham, MA, USA) and the amplicon libraries were assessed using Agilent BioAnalyzer 2100 DNA 1000 chip (Agilent, Santa Clara, CA, USA). For the MiSeq run, 25% PhiX (a bacteriophage at 10 pM) was spiked with a pool of amplicon libraries to enhance the sequencing quality and to minimize crosstalk (Mukherjee et al., 2015), producing 300 bp paired ends with the 600-cycle kit. The raw reads were then deposited in the Sequence Read Archive database under BioProject Number: PRJNA1220023.

### Illumina sequence data processing and statistical analyses

We downloaded a total of 11,303,903 raw fastq sequence files from Basespace and used DAD2 in R studio (version 4.2) to perform microbiome data analysis (Callahan et al., 2016). We first filtered out forward and reverse reads with more than two expected erroneous base calls and less than 175 base pairs. We then removed reads from the PhiX bacteriophage genome from the primer-trimmed Miseq paired-end reads using DADA2 (Callahan et al., 2016). We truncated forward and reverse reads to 250 and 200 base pairs respectively at a median quality score above 30 across samples. Reads were then merged, and chimeras were removed. After quality filtering steps, there were 9,805,562 reads remaining (86.7%) across all samples. SILVA 138.1 16S rRNA gene reference database was used to determine the taxonomies of generated amplicon sequence variants (ASVs) (Quast et al., 2012). A phyloseq object comparable to a classical Operational Taxonomic Unit (OTU)/ASV table was generated by combining count and taxonomy information of generated ASVs and used for diversity analyses in QIIME v.1.8 (Caporaso et al., 2010; Kuczynski et al., 2012). The resulting ASV table was curated by removing reads unclassified at the kingdom domain level, and reads classified as plants, mitochondria, chloroplasts, and archaea. The curated table was then rarefied to 4,651 reads per sample at the family-level prior to diversity analyses (Weiss et al., 2017; Cameron et al., 2021).

Significant differences in Chao 1 (for microbial richness) and Shannon’s Index (for microbial evenness) among comparative groups were assessed using the non-parametric Wilcoxon tests in the event of overall significant difference in the applied non-parametric test in JMP Pro 15 (SAS., Cary, NC, USA). Microbial community composition was assessed using the rarefied ASV table, we assessed microbial diversity assessed using Chao 1 (for richness) and Shannon’s Index (for evenness). Community composition was assessed using Bray-Curtis dissimilarity distance matrix (Bray & Curtis, 1957), which was used to create non-metric multidimensional scales (NMDS) plots to visualize microbial community composition. Permutational multivariate analysis of variance (PERMANOVA) followed by pairwise group comparisons was then used to access the difference across these categorical groups (Anderson, 2017). The differential abundances of ASVs among comparative groups were then analyzed using the group significance command in Quantitative Insights Into Microbial Ecology (QIIME) to identify taxa that may be driving differences in composition determined in the PERMANOVA analysis. This will result in the generation of table for each microbial component in the rarified ASV table, along with the abundance scores across sample categories, the associated Kruskal–Wallis test statistic comparing these categories, and the respective FDR- and Bonferroni-corrected *P* values. Relative abundances were analyzed and plotted only for ASVs that were statistically significant, enabling quantitative comparisons of differentially abundant ASVs across sample categories. All graphs were plotted using R studio (R Core Team, 2023).

## Results

**Figure 1:**
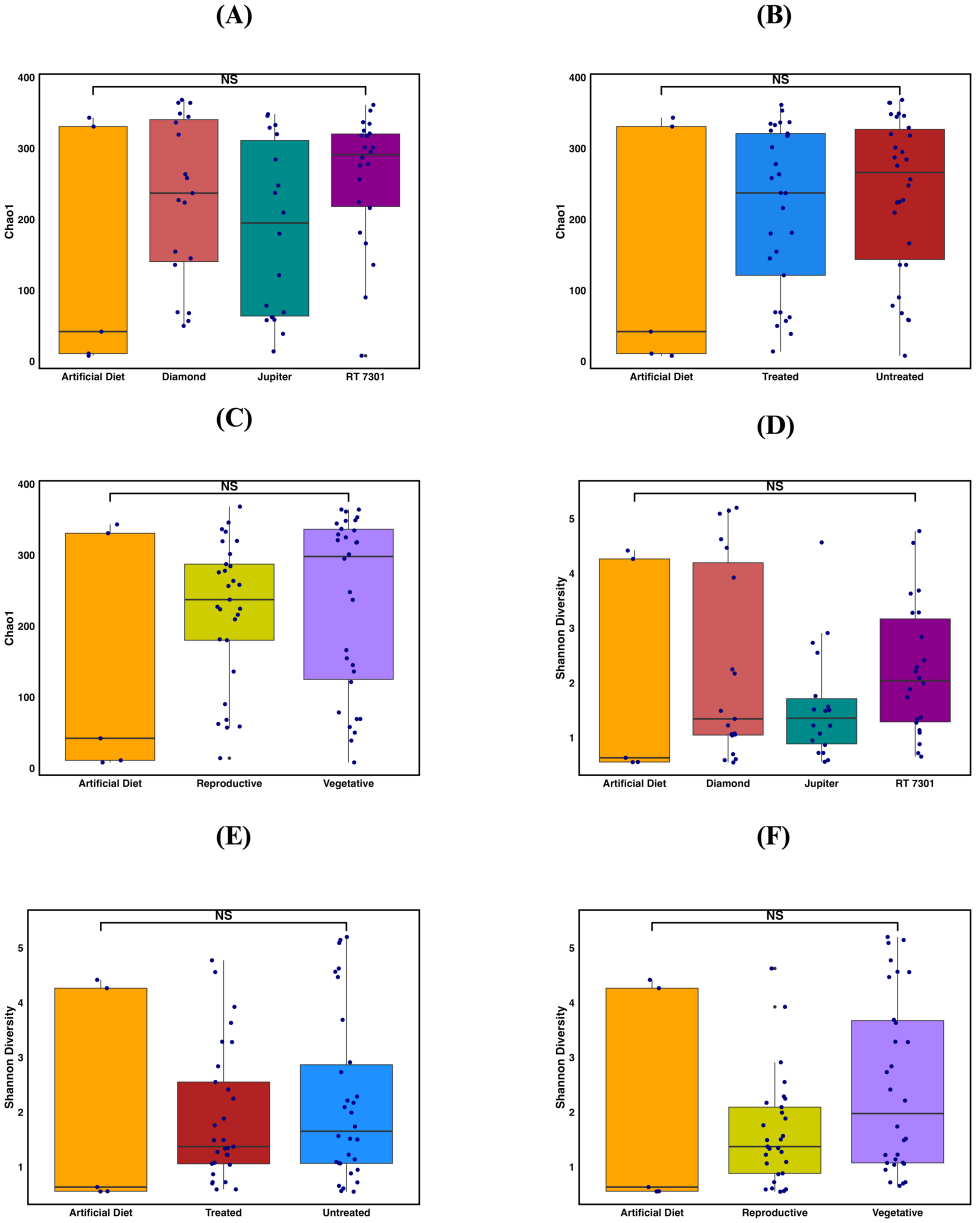
Gut microbiome diversity of FAW larvae. Microbial richness (Chao 1) in Fall armyworm larvae fed on rice across (A) Cultivars (Kruskal–Wallis: *p* = 0.250) (B) Seed Treatment (Kruskal–Wallis: *p* = 0.393), and (C) Phenological stages (Kruskal–Wallis: *p* = 0.339). Microbial evenness (Shannon Index) in Fall armyworm larvae fed on rice across (D) Cultivars (Kruskal–Wallis: *p* = 0.329, (E) Seed Treatment (Kruskal–Wallis: *p* = 0.556), and (F) Phenological stages (Kruskal–Wallis: *p* = 0.135). Diversity estimates were not statistically significant across any of the variables in FAW gut microbiomes.

In the present study, alpha diversity was not significantly different from any of the factors examined in the study. The factors were not significantly differed among each other for both the indices: Chao 1 (Cultivars: Kruskal–Wallis: chi-squared (*χ*^2^)= 4.1039, *df* = 3, *p*-value (P) = 0.250 (Fig. 1A); Treatment: Kruskal–Wallis: (*χ*^2^)= 1.8638, *df* = 2, *P* = 0.393 (Fig. 1B); and Phenological stage- Kruskal–Wallis: (*χ*^2^)= 2.1615, *df* = 2, *P* = 0.339 (Fig. 1C) and Shannon diversity (Cultivars- Kruskal–Wallis:(*χ*^2^)= 3.4315, *df* = 3, *P* = 0.329; Fig. 1D; Treatment- Kruskal–Wallis: (*χ*^2^)= 1.1731, *df* = 2, *P* = 0.556 (Fig. 1E); and Phenological stage- Kruskal–Wallis: (*χ*^2^) =4.005, *df* = 2, *P* = 0.135 = Fig. 1F).

**Figure 2:**
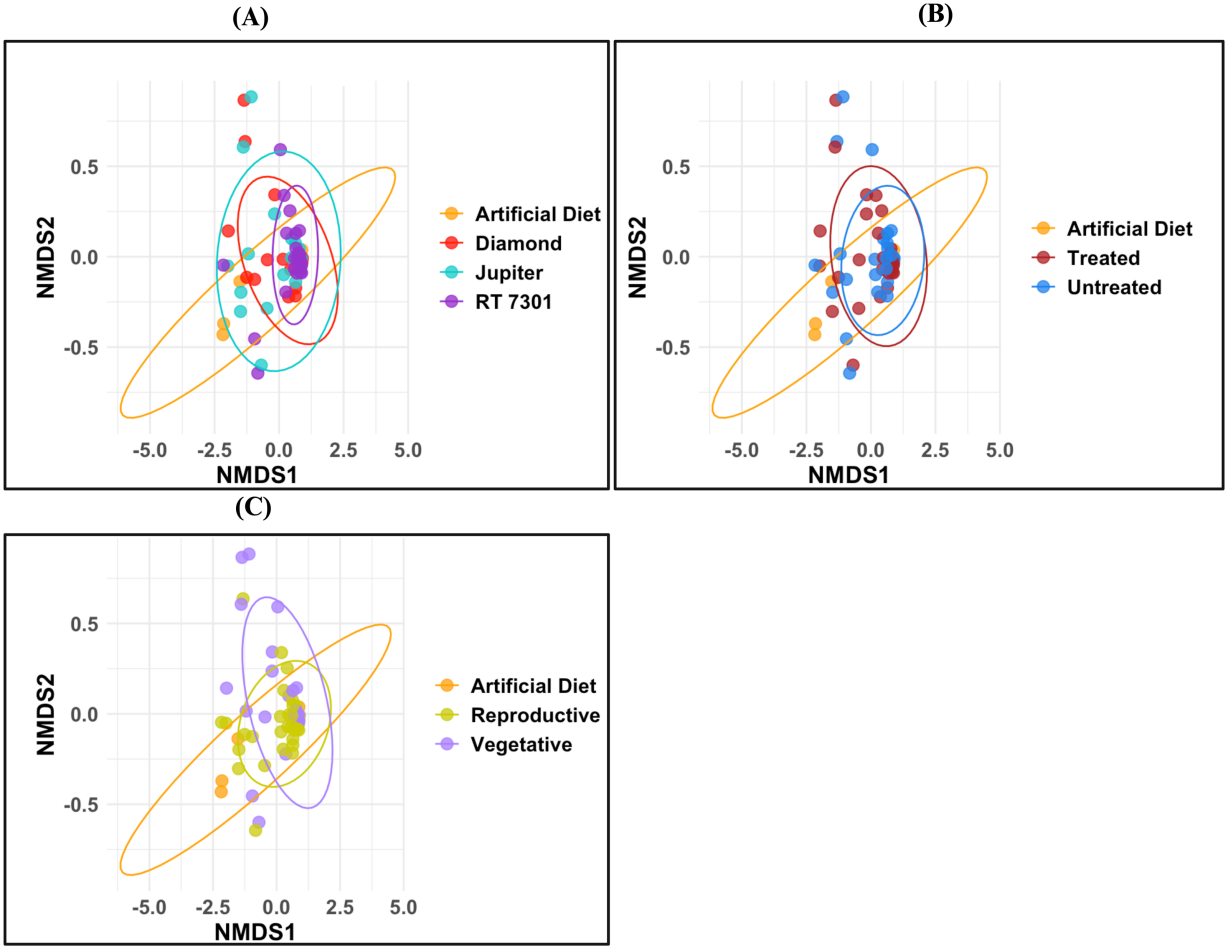
Community composition of FAW larvae. Non-metric multidimensional scaling (NMDS) plots showing the variations in microbiome composition of Fall armyworm fed on rice across (A) Cultivars (PERMANOVA: *p* = 0.557), (B) Seed Treatments (PERMANOVA: *p* = 0.703), and (C) Phenological stages (PERMANOVA: *p* = 0.004). There were no statistically significant differences on microbial community composition in FAW larvae across the examined variables.

**Figure 3:**
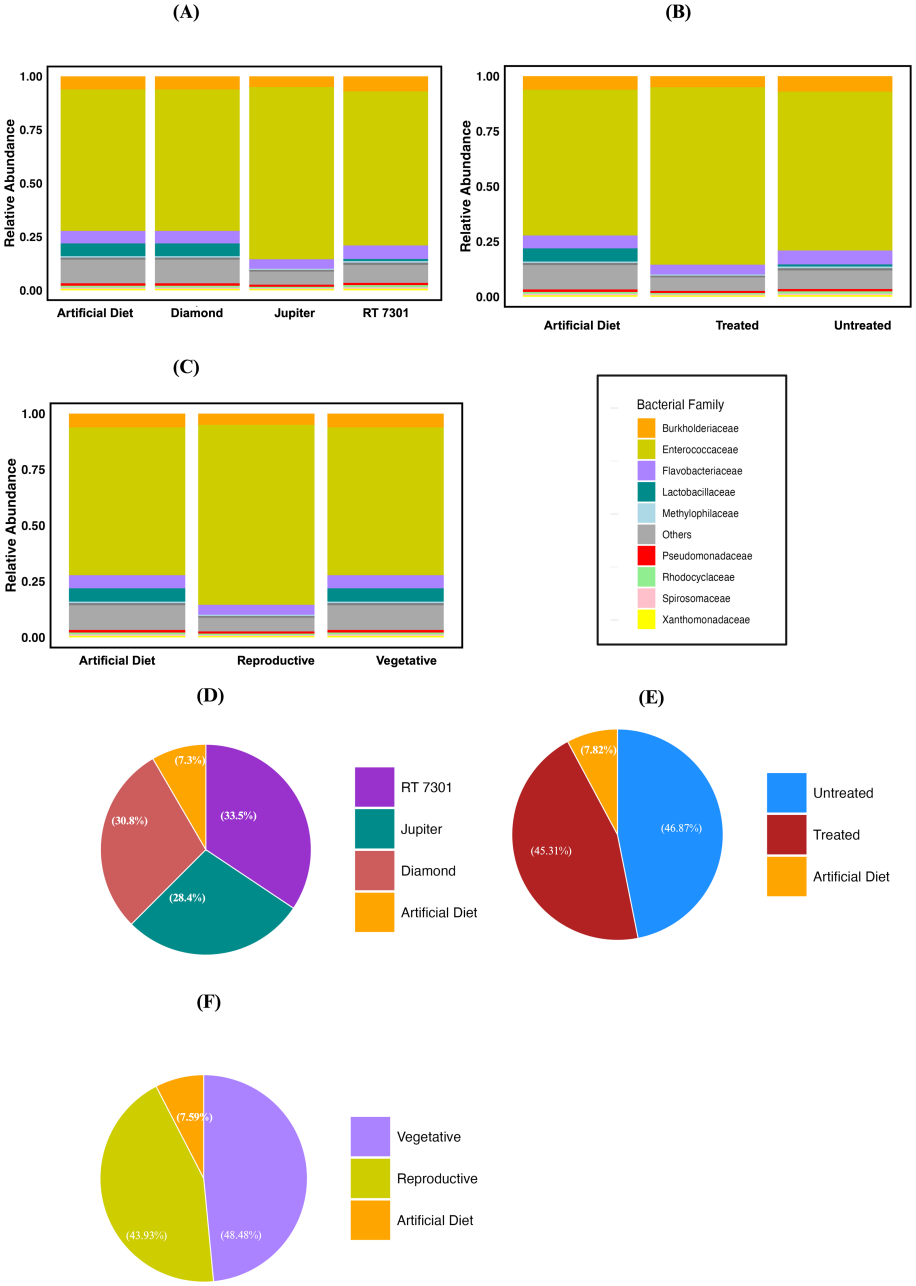
Relative abundances of bacterial families in FAW larvae. Relative abundances of bacterial families that are significantly different (*p* = 0.05) in fourth instar FAW larvae fed on rice across (A) Cultivars (B) Seed Treatments, and (C) Phenological stages, as well as the percentage distributions of these across (D) Cultivars, (E) Seed Treatments, and (F) Phenological stages.

Similarly, we failed to find any differences in the gut bacterial composition between the selected cultivars (PERMANOVA: *F* = 0.83, R-squared: 0.038 *p* = 0.557, Fig. 2A), and seed treatment (PERMANOVA: *F* = 0.607, R-squared=0.018, *p* = 0.703, Fig. 2B). However, the gut microbiomes of FAW larvae fed on phenological stages showed distinct dissimilarities (PERMANOVA: *F* = 3.89, R-squared = 0.109, *P* = 0.004, Fig. 2C; Tables S1, S2). Additionally, we also failed to detect any difference across cultivars and treatment, even after removing the artificial diet (control) from the analysis. Hence, our study revealed that crop phenology was the only critical factor impacting FAW larval gut microbiota and the comparison of gut bacterial communities associated with FAW feeding showed differences across bacterial families. The most abundant OTU associated with FAW irrespective of cultivars (Fig. 3A), seed treatment (Fig. 3B), and phenological stages (Fig. 3C) belongs to family *Enterococccaceae* (60–80%), followed by *Burkholdericaeae* (6–7%). The relative abundance of microbial taxa varies across cultivars, with the highest in RT 7301 (34.37%), Diamond (29.12%), Jupiter (28.12%), and the lowest in artificial diet (7.81%) (Fig. 3D). Among the seed treatments, treated (45.31%), had the slightly lowest microbiota compared to untreated (46.87%) (Fig. 3E) and across the phenological stages, vegetative had the highest (48.48%), compared to reproductive stage (43.93%) (Fig. 3F).

## Discussion

Among the insect herbivores, FAW is among the top 10 most damaging insects in the world to staple food crops including rice, thus jeopardizing food security (Li et al., 2022). Insect gut microbiomes play a major role in insects in overall development and survival (Jing, Qi & Wang, 2020). Though rice is considered a minor host in the United States, the likelihood of being a major defoliator is imminent due to drastic changes in global temperature (Yan et al., 2022). Functionally, FAW gut microbiomes contribute significantly to degrading and detoxifying a wide range of insecticides, further enhancing their pervasive status (Zhang et al. 2024a; Zhang, Zhang & Xu, 2023; Siddique et al., 2025). Previous studies have shown that insect gut microbiota is shaped by insect developmental stages, the environment, and the diet they consume (Li et al., 2022). However, the role of host plant phenology in shaping the gut microbiota of insects (potentially minimizing their functions and making FAW control and management more feasible) is poorly understood.

Our study assessed the microbial diversity and composition of FAW larval gut microbiomes fed on different rice cultivars under seed treatment at two phenological stages. Our prior studies demonstrated the differential response of FAW larvae across rice phenology and seed treatment by a short-term exposure of 48 h (Balakrishnan et al., 2025). Here, we found that the FAW larval survival rate was lower when they fed on vegetative stages compared to reproductive stages and seed-treated plants. Based on these results, we speculated that FAW larval gut microbiomes would vary across phenology, cultivars and seed treatment. Contrary to our expectations, in the current study, we found FAW larval gut microbial community composition was primarily shaped by rice crop phenology (Fig. S1) and not by cultivars, or seed treatment. The 96-hour feeding time used is comparable to other studies using FAW larvae that even less (48 h) feeding period, found significant changes on the development of FAW (Vasquez et al., 2024; Balakrishnan et al., 2025). However, in this study, we speculate that the 96-hour feeding time might have also influenced our results, and additional experiments should allow continuous feeding-based assays.

The importance of understanding the response of FAW towards host phenology has already been validated by our prior studies. Vasquez et al. (2024) demonstrated the importance of phenology in sorghum–sudangrass–FAW interactions, with the herbivore attaining the lowest mass, longest pupation time and lowest pupal mass in late stages (booting stage), when compared to early stages (3-leaf and panicle inflation stages). Similarly, Ayala et al. (2024) provided additional evidence by confirming the role of phenology in soybean–FAW interactions. They observed the highest mortality of FAW on mid-stage (R3) compared to early (V3) and late stages (R7). In the current study, we observed the highest microbial composition in the vegetative stage than the reproductive stage. The variation in bacterial diversity may be related to differences in toxin and metabolite concentrations between the vegetative and reproductive stages in accordance with the defense fitness hypothesis (Watts, Kaur & Kariyat, 2023). In our previous study (Balakrishnan et al., 2025), we quantified the surface defenses, particularly trichomes and wax in rice. We found that both trichome density and wax content were substantially higher in the vegetative stage than the reproductive stage corroborating our earlier conclusion that surface defenses are phenology dependent. We speculate lowered defenses during the reproductive stage may result from resource allocation costs related to flowering and seed production. This may be driving the observed differences in microbial community composition between these two phenological stages.

Among different gut microbiota, *Enterococcaceae* was the most abundant bacterial family across all the samples regardless of the treatments. Previous studies have shown that *Enterococcaceae* belonging to the phylum Firmicutes have a major role in providing tolerance to toxic diets, in addition to the synthesis of essential vitamins and pheromones and are the core members of microbial community in FAW (Li et al., 2022; Gomes, 2024). For instance, Chen et al. (2022), reported that *Enterococcus* sp. improves growth traits by enhancing nutrient utilization. Similar to our findings, Wu, Hu & Liu (2024) also reported the highest microbial load of Enterococcus in FAW larvae, with its presence detected in individuals fed on different host plants (pepper, tomato, maize, and sorghum). Furthermore, Almeida et al. (2017) observed the role of *Enterococcaceae* (*E. casseliflavus* and *E. mundtii)* in degrading and detoxifying insecticides such as chlorpyrifos ethyl, lambda-cyhalothrin and Spinosad. Similar species were also detected in FAW fed on Bt and antibiotics suggesting their ability to survive under altered gut conditions (Castañeda-Molina et al., 2023). Moreover, *E. mundtii*, a species associated with *Spodoptera littoralis*, has also been reported to secrete antimicrobial peptides that selectively kill *Bacteroides* spp. eradicating the pathobionts in the gut (Basit et al., 2025). Hence, these studies collectively highlight the importance of *Enterococcus* sp. in the survival and adaptation of FAW. In addition to *Enterococcaceae*, other bacterial families such as *Burkholderaceae*, *Flavobacteriaceae, Lactobacillaceae,* and *Pseudomonadaceae* were also observed in all the samples. These bacterial families have also been proven to be involved in the various functions that collectively enhance the adaptability and survival of insects (Bansal et al., 2014; Shao, Mason & Felton, 2024; Herrera-Cardoso et al., 2025). For instance, Rogowska-van der Molen et al. (2023) reported the ability of *Pseudomonas sp.* (Pseudomonadaceae) to detoxify the plant toxin, Nitropropionic acid (in leguminous crops) in the gut of Southern green shield bug *Nezara viridula*. However, future studies investigating the species level identification of microbes and their roles in FAW are essential in improving pest management strategies.

In conclusion, this study provides the promising evidence of supporting the importance of phenology in determining the microbial load in insects. Further studies should also explore and perform a comprehensive evaluation on the specific microbial families that are closely associated with FAW by screening different concentrations of antibiotics and their effect on FAW fitness (Lin et al., 2015). In addition, the role of rice secondary metabolites and defenses against FAW essential microbes is also an area of interest. This will assist in developing sustainable eco-friendly pest management strategies by using microbes as biocontrol agents or by generating genetically modified microbes that impact the herbivore health and counteract the detoxification mechanisms (Haider et al., 2025).

## Supplemental Information

10.7717/peerj.20458/supp-1 Supplemental Information 1 Statistical table of gut bacterial diversity and composition of FAW analysis Summarized statistical table of gut bacterial diversity and composition in fall armyworm fed on different rice cultivars under seed treatment at vegetative and reproductive growth stages

10.7717/peerj.20458/supp-2 Supplemental Information 2 Pairwise comparison data from PERMANOVA analysis of gut bacterial diversity Pairwise comparison data from PERMANOVA analysis of gut bacterial diversity and composition in fall armyworm fed on different rice cultivars under seed treatment at vegetative and reproductive growth stages

10.7717/peerj.20458/supp-3 Supplemental Information 3 Relative abundances of bacterial communities in FAW Relative abundances of bacterial (A) Phylum, (B) Order, and (C) Class that are significantly different (*P* = 0.05) in fourth instar FAW larvae fed on rice across phenological stages

## Additional Information and Declarations

### Data Availability

The following information was supplied regarding data availability: The raw sequence data are available at NCBI SRA BioProject: PRJNA1220023.
