# Supplementary material for: Phenology overshadows seed treatment and cultivar effects on fall armyworm gut microbiome following short-term feeding on rice
Source: PeerJ. 2026 Jan 20;14:e20458. doi: 10.7717/peerj.20458 (PMC12829461; doi:10.7717/peerj.20458)
Supplement: Supplemental Information 1 — Summarized statistical table of gut bacterial diversity and composition in fall armyworm fed on different rice cultivars under seed treatment at vegetative and reproductive growth stages [file peerj-14-20458-s001.docx]

**Table S1:** Summarized statistical table of gut bacterial diversity and composition in fall armyworm fed on different rice cultivars under seed treatment at vegetative and reproductive growth stages

| Response Variable | Factors | Statistical Test | P value |
| --- | --- | --- | --- |
| **Alpha Diversity** | | | |
| 1. Chao1 | Cultivar | Kruskal-Wallis: (χ²) = 4.1039, df = 3 | 0.250 |
|  | Seed Treatment | Kruskal-Wallis: (χ²) = 1.8638, df = 2 | 0.394 |
|  | Phenological stage | Kruskal-Wallis: (χ²) = 2.1615, df = 2 | 0.339 |
| 1. Shannon | Cultivar | Kruskal-Wallis: (χ²) = 3.4315, df = 3 | 0.329 |
|  | Seed Treatment | Kruskal-Wallis: (χ²) = 1.1731, df = 2 | 0.556 |
|  | Phenological stage | Kruskal-Wallis: (χ²) = 4.005, df = 2 | 0.135 |
| **Beta Diversity** | Cultivar | PERMANOVA: F=0.83, R-squared: 0.038 | 0.557 |
|  | Seed Treatment | PERMANOVA: F= 0.60, R-squared=0.018 | 0.703 |
|  | Phenological stage | PERMANOVA: F=3.89, R-squared=0.109 | 0.004 |
