# Supplementary material for: Phenology overshadows seed treatment and cultivar effects on fall armyworm gut microbiome following short-term feeding on rice
Source: PeerJ. 2026 Jan 20;14:e20458. doi: 10.7717/peerj.20458 (PMC12829461; doi:10.7717/peerj.20458)
Supplement: Supplemental Information 2 — Pairwise comparison data from PERMANOVA analysis of gut bacterial diversity and composition in fall armyworm fed on different rice cultivars under seed treatment at vegetative and reproductive growth stages [file peerj-14-20458-s002.docx]

Table S2: Pairwise comparison data from PERMANOVA analysis of gut bacterial diversity and composition in fall armyworm fed on different rice cultivars under seed treatment at vegetative and reproductive growth stages

| Pairwise | F-value | R-squared | P value |
| --- | --- | --- | --- |
| Artificial Diet vs Vegetative | 0.2012 | 0.005717 | 0.779 |
| Artificial Diet vs Reproductive | 1.1907 | 0.035876 | 0.316 |
| Vegetative vs Reproductive | 5.1935 | 0.080904 | 0.019 |
