# Supplementary figures and images for: Phenology overshadows seed treatment and cultivar effects on fall armyworm gut microbiome following short-term feeding on rice
Source: PeerJ. 2026 Jan 20;14:e20458. doi: 10.7717/peerj.20458 (PMC12829461; doi:10.7717/peerj.20458)

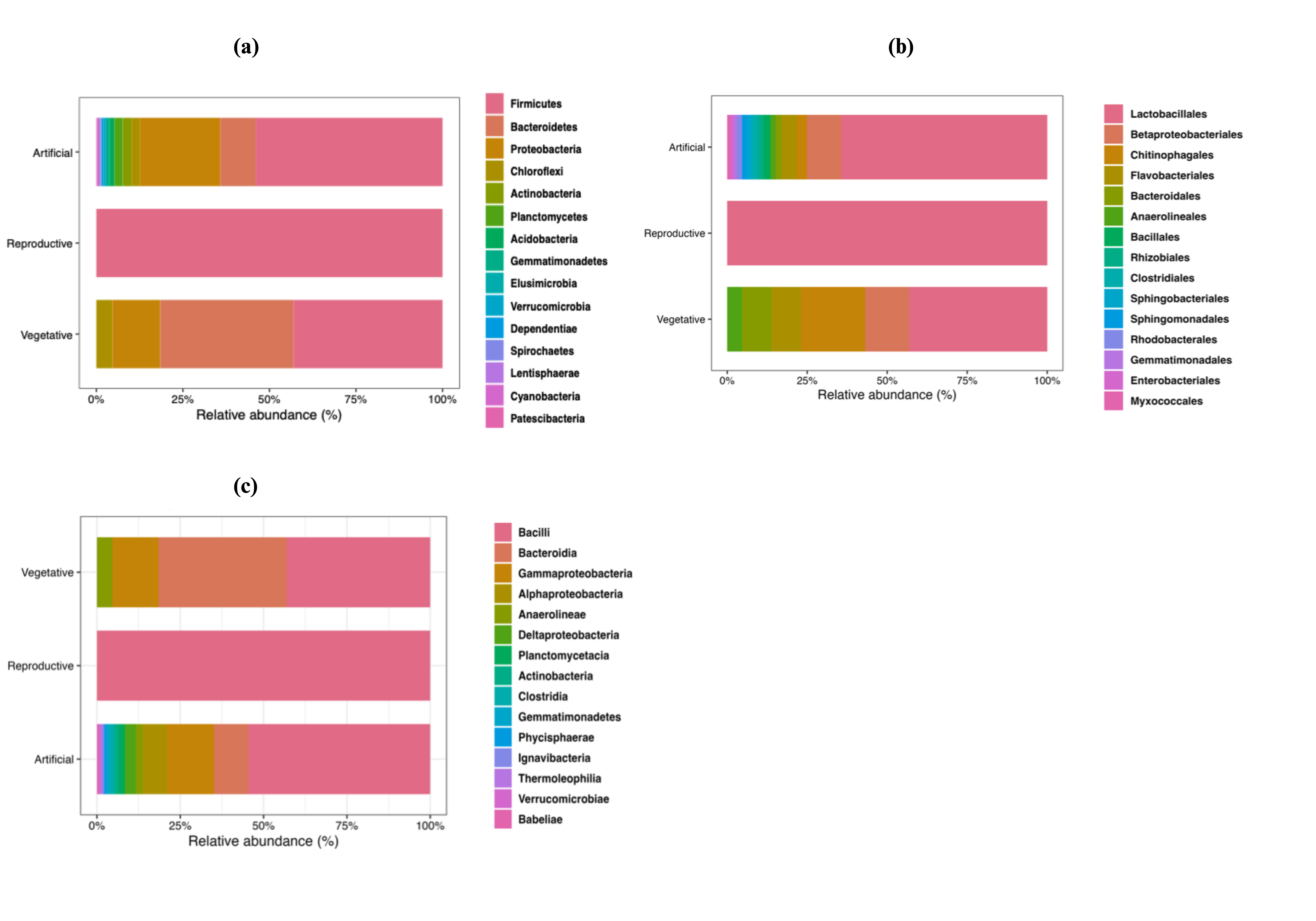

Supplement: Supplemental Information 3 — Relative abundances of bacterial (A) Phylum, (B) Order, and (C) Class that are significantly different (P = 0.05) in fourth instar FAW larvae fed on rice across phenological stages [file peerj-14-20458-s003.png]
